# Unravelling the enigma of selective vulnerability in neurodegeneration: motor neurons resistant to degeneration in ALS show distinct gene expression characteristics and decreased susceptibility to excitotoxicity

**DOI:** 10.1007/s00401-012-1058-5

**Published:** 2012-11-13

**Authors:** Alice Brockington, Ke Ning, Paul R. Heath, Elizabeth Wood, Janine Kirby, Nicolò Fusi, Neil Lawrence, Stephen B. Wharton, Paul G. Ince, Pamela J. Shaw

**Affiliations:** 1 Academic Neurology Unit, Sheffield Institute for Translational Neuroscience (SITraN), University of Sheffield, 385A Glossop Road, Sheffield, S10 2HQ UK; 2 Academic Neuropathology Unit, Sheffield Institute for Translational Neuroscience (SITraN), University of Sheffield, 385A Glossop Road, Sheffield, S10 2HQ UK; 3 Computational Biology Unit, Sheffield Institute for Translational Neuroscience (SITraN), University of Sheffield, 385A Glossop Road, Sheffield, S10 2HQ UK

## Abstract

A consistent clinical feature of amyotrophic lateral sclerosis (ALS) is the sparing of eye movements and the function of external sphincters, with corresponding preservation of motor neurons in the brainstem oculomotor nuclei, and of Onuf’s nucleus in the sacral spinal cord. Studying the differences in properties of neurons that are vulnerable and resistant to the disease process in ALS may provide insights into the mechanisms of neuronal degeneration, and identify targets for therapeutic manipulation. We used microarray analysis to determine the differences in gene expression between oculomotor and spinal motor neurons, isolated by laser capture microdissection from the midbrain and spinal cord of neurologically normal human controls. We compared these to transcriptional profiles of oculomotor nuclei and spinal cord from rat and mouse, obtained from the GEO omnibus database. We show that oculomotor neurons have a distinct transcriptional profile, with significant differential expression of 1,757 named genes (*q* < 0.001). Differentially expressed genes are enriched for the functional categories of synaptic transmission, ubiquitin-dependent proteolysis, mitochondrial function, transcriptional regulation, immune system functions, and the extracellular matrix. Marked differences are seen, across the three species, in genes with a function in synaptic transmission, including several glutamate and GABA receptor subunits. Using patch clamp recording in acute spinal and brainstem slices, we show that resistant oculomotor neurons show a reduced AMPA-mediated inward calcium current, and a higher GABA-mediated chloride current, than vulnerable spinal motor neurons. The findings suggest that reduced susceptibility to excitotoxicity, mediated in part through enhanced GABAergic transmission, is an important determinant of the relative resistance of oculomotor neurons to degeneration in ALS.

**Electronic supplementary material:**

The online version of this article (doi:10.1007/s00401-012-1058-5) contains supplementary material, which is available to authorized users.

## Introduction

Amyotrophic lateral sclerosis (ALS) is an adult-onset neurodegenerative disorder, in which there is selective loss of motor neurons in the cerebral cortex, brainstem and spinal cord. Symptoms often begin focally but with progression of disease, there is spread of weakness to the limb, bulbar and respiratory muscles. Mean survival from onset is 3 years. On pathological examination, there is loss of lower motor neurons in the ventral horn of the spinal cord, with astrocytic gliosis, and the presence of ubiquitinated inclusions in surviving motor neurons. An upper motor neuron axonopathy is demonstrated by myelin pallor and gliosis of the corticospinal tracts, which may be accompanied by a reduction in cortical pyramidal neurons [17]. Approximately 5 % of ALS cases are familial, and 70 % of these have mutations in *C9ORF72*, *SOD1*, *TDP43* or *FUS*. In the majority of ALS cases, the cause of motor neuron degeneration is unknown, although a number of pathogenic processes, including excitotoxicity, oxidative stress, protein aggregation, mitochondrial dysfunction, dysregulation of the cytoskeleton and axonal transport, and inflammation are considered to play important roles [9].

A consistent clinical feature of the disease is the sparing of eye movements and the function of external sphincters, so that patients retain continence. Pathological studies have confirmed that there is relative sparing of the cranial motor nuclei of the oculomotor, trochlear and abducens nerves, and of Onuf’s nucleus in the sacral spinal cord, which innervates the external sphincters of the pelvic floor [36]. Although neuronal numbers are relatively well-preserved in these resistant motor nuclei, pathological changes resembling those seen in anterior horn cells are present to a lesser degree [40, 41]. The earliest and most severe changes in ALS affect motor neurons, but there is increasing recognition of variable clinical and pathological involvement outside the motor system. Fronto-temporal dementia (FTD), or more subtle cognitive deficits may affect patients with ALS [27], with ubiquitinated inclusions in the dentate granule cells and neocortex [33]. Other long tracts, including the dorsal column and spinocerebellar tracts [17] show degenerative changes in some patients. Patients who are treated with ventilation for a prolonged period after the onset of respiratory failure may develop more widespread CNS degeneration including, in some cases, loss of oculomotor neurons on neuropathological examination [13]. 

Thus ALS is a degenerative disease, in which motor neurons are relatively selectively vulnerable, but other neuronal subgroups are affected in some patients, particularly after a prolonged disease course. Two groups of lower motor neurons: the oculomotor nuclei, and the sacral nucleus of Onuf, are relatively resistant to the disease process. Rodents which overexpress mutant human SOD1 develop motor neuron degeneration, and in this animal model of ALS, oculomotor nuclei are also relatively spared [38]. Studying the differences in properties of neurons that are vulnerable and those that are resistant to the disease process in ALS may provide insights into the mechanisms of neuronal degeneration, and identify targets for therapeutic manipulation.

The oculomotor nucleus innervates four of the six extraocular muscles (EOMs), which display a distinct phenotype, gene expression profile [49] and disease responsiveness [47]. They have a unique composition of six fibre types, distinct from other skeletal muscles, some with very high mitochondrial content and marked fatigue resistance. Such differences may be determined by their distinct embryonic origin, or by demands imposed by the relative complexity of oculomotor control systems, and the specific discharge patterns of oculomotor neurons [46]. Motor unit discharge patterns are a key determinant of skeletal muscle properties, and EOMs and oculomotor neurons interact in a highly specific manner: explants of neonatal extraocular muscle grown in co-culture with the incorrect spinal motor neurons die faster than those grown with oculomotor neurons [48]. The pattern of innervation of EOMs is different from other skeletal muscles. Neuromuscular junctions are distributed throughout the fibre length at a higher density [12], and show some structural differences [23]. 20 % of EOM fibres are innervated by multiple neuromuscular junctions [44]. Oculomotor motor units are amongst the smallest seen in any skeletal muscle, [47], with higher maximum motor neuron discharge rates. Even in the primary position of gaze, 70 % of oculomotor neurons are active, commonly discharging at 100 Hz [50]. This level of activity would be predicted to place a significant metabolic demand on the neuron.

We have used microarray analysis to compare the gene expression profiles of isolated motor neurons from the ALS-resistant oculomotor nucleus and ALS-vulnerable spinal cord in post-mortem tissue from four neurologically normal control subjects. We show that motor neuron subtypes with different susceptibility to the disease process in ALS also have markedly different transcriptional profiles. Oculomotor (OM) and lumbar spinal cord (LSC) motor neurons show differential expression of genes with a function in synaptic transmission, ubiquitin-dependent proteolysis, mitochondrial function, transcriptional regulation, immune system functions, and the extracellular matrix. The most striking differences are seen in genes with a function in synaptic transmission, in particular GABA and glutamate receptor subunits. We have validated these observed changes, using unpublished transcriptional data from the oculomotor nucleus and spinal cord of two other species, obtained from the public repository GEO Omnibus. Furthermore, we demonstrate the functional impact of altered expression of GABA and glutamate receptors on whole-cell currents induced by agonists at GABA and glutamate receptors.

## Materials and methods

### Case selection

Brain and spinal cord tissue from four neurologically normal human control subjects (Table 1) was obtained from the Sheffield Brain Tissue Bank. Tissue donated for research was obtained with informed consent from the next of kin, and in accordance with the Human Tissue Authority guidelines on tissue donation. Midbrain and lumbar spinal cord sections were collected postmortem, processed according to standard protocols [18], and stored at −80 °C until required. No abnormality of the midbrain or spinal cord was found at postmortem on full neuropathological examination.

**Table 1:** Characteristics of cases

Case number	Sex	Age at death	Cause of death	Postmortem delay time (h)
1	M	64	Ischaemic heart disease	12
2	M	64	Empyema	30
3	F	59	Pneumonia	5
4	M	67	Hepatocellular carcinoma	63

### Tissue preparation and laser capture microdissection

Frozen tissue sections were mounted in Cryo-M-Bed embedding compound (Bright UK), and 10 μm sections cut in a cryostat at −22 °C. Frozen sections were thaw mounted onto slides at room temperature, fixed in 70 % ethanol, washed in DEPC-treated water, and stained for 1 min in a solution of 0.1 % w/v Toluidine Blue in 0.1 M sodium phosphate. They were then washed and dehydrated through graded ethanol concentrations (70, 90 and 100 %), and xylene. OM and LSC motor neurons, identified by staining, anatomical location, size and morphology, were isolated on Capsure Macro LCM caps using the Arcturus PixCell II laser capture microdissection system (Arcturus Bioscience). Approximately 800 successful laser capture firings were made from the spinal cord and 800 from the OM nucleus of each case (supplementary figure 1). The procedure of laser capture microdissection does not prevent the collection of small amounts of adjacent neuropil and glial cells, but significantly enriches the extracted RNA for motor neuron-expressed transcripts.

### RNA isolation and amplification, and Affymetrix GeneChip processing

From each sample >50 ng RNA was extracted using the PicoPure™ RNA isolation kit (Arcturus), and amplified using a 2-cycle linear amplification process, with the GeneChip two-cycle target labelling and control kit (Affymetrix), and MEGAscript^®^ T7 kit (Ambion). The linear amplification technique has been shown to generate highly reproducible gene expression profiles from small starting quantities of RNA [32]. This procedure produced 50–100 μg of biotin-labelled antisense RNA for each sample. 15 μg amplified cRNA was fragmented by heating to 94 °C for 20 min, and spiked hybridization controls were added. Each sample was hybridized to one GeneChip Human Genome U133 Plus 2.0 Array (Affymetrix) and scanned in the GeneChip Scanner 3000 to detect fluorescent hybridization signals. We carried out quality control (QC) measures, according to Affymetrix protocols, to ensure that RNA was of sufficient quality and was matched between samples. CEL files generated by the Affymetrix GeneChip Operating System were taken forward for microarray data analysis.

### Microarray data analysis

We wanted to detect differentially expressed transcripts between the group of oculomotor samples and the group of spinal cord samples. The bioconductor^1^ [10] package PUMA [45] was used to carry out the normalization and differential expression analysis. PUMA is a Bayesian probabilistic model for probe-level analysis that takes into account the probe-level measurement error while estimating the gene expression levels. This measurement error is propagated downstream into the analysis, for instance when detecting differentially expressed transcripts This has been shown to increase the accuracy in detecting differential expression, making the model more resistant to outliers even at small sample sizes [29]. The output of this procedure is the posterior probability (PPLR value), estimated using Bayesian inference, of a transcript’s differential expression in the group of oculomotor samples and the group of spinal cord samples. Since this computation is essentially a univariate test, care must be taken to account for multiple hypotheses testing. For this reason, we computed *q* values from the posterior probabilities as suggested in [52]. Genes that were significantly (*q* value less than 0.001) differentially expressed were assigned Gene Ontology (Gene Ontology project; http://www.geneontology.org/ [1]) and Kegg Pathway (Kyoto Encyclopaedia of Genes and Genomes; http://www.genome.jp/kegg/ [20]) annotations, and gene ontology (GO) enrichment analysis was performed using DAVID software (NIAID/NIH; http://david.abcc.ncifcrf.gov/summary.jsp, [6]).

### Analysis of differential expression of oculomotor nucleus and spinal cord tissue of rat and mouse

Two datasets were obtained from the Gene Expression Omnibus public functional genomics data repository (http://www.ncbi.nlm.nih.gov/geo/). Dataset GSE3305 analysed total RNA extracted from OM nucleus and spinal cord of rats at 6, 18 and 30 months of age, using TRIzol. Tissues from four animals were combined in each RNA sample. Biotinylated RNA samples were hybridized to rat RA230 Affymetrix microarray chips (*n* = 18). Dataset GSE3343 analysed RNA extracted from the oculomotor nucleus and spinal cord of mice at 10 weeks of age, hybridized to mouse 430A Affymetrix microarray chips (*n* = 6). The data were normalized, and probe-level analysis conducted using MAS5 software, and made freely available to the scientific community. For the purposes of the present study, as there were relatively few significant differences in the gene expression profile between the three rat age groups, the data from 6, 18 and 30 months were combined. As the .CEL files were unavailable in the repository for PUMA analysis, differential expression between oculomotor and spinal tissue was determined using an unpaired *t* test.

### Quantitative PCR

RNA extracted from laser-captured OM and LSC motor neurons as above, which was not required for hybridization to microarray chips, was taken forward for use in quantitative PCR to verify expression levels of GABRA1. cDNA was synthesized using Superscript II reverse transcriptase, according to manufacturer’s protocol (Invitrogen). QPCR was performed using 12.5 ng cDNA, 1× SYBR Green PCR master mix (Applied Biosystems), 900 nM forward primer (CCTTCCAGACTTCTCATGGCTAAC) and 600 nM reverse primer (TAGCAGGAAGCAGACTAATAAGAAATATTC), to a total volume of 20 μl. After an initial denaturation at 95 °C for 10 min, templates were amplified by 40 cycles of 95 °C for 15 s and 60 °C for 1 min, on an MX3000P Real-Time PCR system (Stratagene). Gene expression values, calculated using the ΔΔ*C*_t_ calculation (ABI PRISM 7700 Sequence Detection System protocol; Applied Biosystems), were normalized to β-actin expression, and statistical analysis performed using an unpaired *t* test.

### Preparation of acute spinal cord and midbrain slices for patch clamp recording

Adult male Sprague-Dawley rats were anaesthetized with sodium pentobarbital (50 mg kg^−1^) and decapitated according to the UK Animal (Scientific Procedures) Act 1986 guidelines. Brainstem was isolated, glued on its rostral end to the stage of a vibroslicer, and sliced from the caudal end to the midbrain region of OM nucleus, identified by anatomical landmarks. Two or three 300-μm-thick transverse slices through the OM nucleus were obtained per preparation. The spinal cord was isolated, and 300 μm transverse sections prepared from the lumbar limb expansion, using a McIlwain tissue chopper. Slices were maintained in continuously bubbled (95 % O_2_/5 % CO_2_) bicarbonate buffered saline for at least 1 h prior to recordings.

### Electrophysiology

Whole-cell electrophysiological experiments were recorded as previously described [39]. The location of electrode placement for OM neuron recording is shown in supplementary figure 2. The constituents of all buffers used are detailed in the supplementary experimental procedures. Voltage clamp recordings were performed using an Axon Multi-Clamp 700B amplifier (Axon Instruments) using unpolished borosilicate pipettes placed at the cell soma. Pipettes had a resistance of 2–4 MΩ when filled with intracellular solution. Pipettes filled with high concentrations of Cl^−^ for GABA-induced current recordings were used to maintain the Cl^−^ equilibrium potential close to 0 mV, thereby facilitating the observation of GABAR-mediated whole-cell currents at resting potentials. Cs^+^ in the pipette solution would block K^+^-dependent membrane conductance. Cells were accepted for study if a stable seal formed with a whole-cell resistance of at least 120 MΩ and a series resistance of <10 MΩ. Receptors were activated by focal perfusion of agonists from a micropipette with its tip located 30–50 μm from the cell. Three cells were used for dose–response recordings for AMPA (5 μM to 5 mM) or kainate (50 μM to 50 mM)-induced whole-cell currents in OM and LSC motor neurons. Currents were recorded in extracellular perfusion buffer with 20 mM extracellular Na^+^ at −60 mV. Na^+^ was reduced from 125 mM in normal extracellular solution to 20 mM to reduce the driving force for agonist-evoked current. 100 μM AMPA and 1 mM kainate, which were close to the EC_50_ from the dose–response recordings, were used to measure Ca^2+^ permeability of AMPA receptors in six cells per agonist, in Na^+^-free extracellular solutions containing 50 mM Ca^2+^. Three cells were used for dose–response recording for GABA (0.1–100 mM)-induced whole-cell currents in OM and LSC motor neurons. 10 mM GABA, which was close to the EC_50_ of the dose–response recordings, was used for repeat recording in six cells. All extracellular solutions were supplemented with MK-801 (10 μM), tetrodotoxin (1.0 μM), and Cd^2+^ (100 μM) to block NMDA receptors, voltage-gated Na^+^ channels, and Ca^2+^ channels, respectively. Cells were held at a membrane potential of −60 mV. All recordings were performed at room temperature 21 °C. Current recordings were sampled onto an IBM-PC-compatible computer using pClamp10 software (Axon). Data were filtered at 3 kHz and sampled at 20–40 kHz.

## Results

### Overall patterns of differential gene expression

**Fig. 1:**
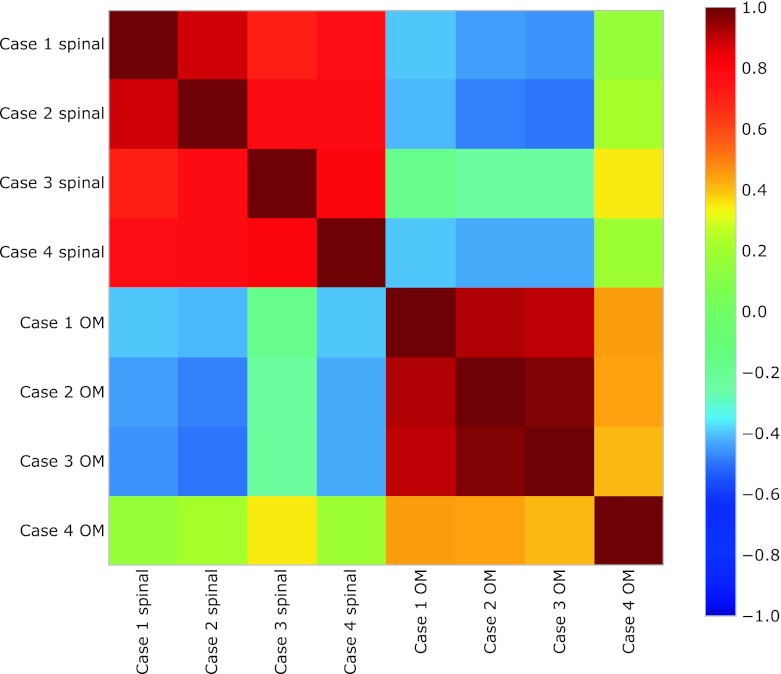
Covariance matrix for the gene expression dataset, evaluated using only the top 100 differentially expressed genes. The samples on both axes are ordered according to the tissue type. The two visible blocks correspond to the two groups, indicating that the most differentially expressed genes are able to capture the difference between oculomotor and spinal cord samples. *Each square* in the plot shows the correlation between each pair of samples. *Warm colours* correspond to a high positive correlation, while *cold colours* correspond to a high negative correlation

A total of 1,521 unique named genes were upregulated in OM neurons, and 236 in LSC motor neurons, at a significance level of *q* < 0.001, which was the threshold used for downstream analysis (Table 2). OM and LSC motor neurons cluster according to their gene expression profiles, as shown in the covariance plot (Fig. 1).

**Table 2:** Numbers of differentially expressed probe sets and PPLR values at different thresholds of *q*

*q* value threshold	Upregulated in oculomotor neurons		Upregulated in spinal motor neurons	
	No. of probe sets	PPLR values	No. of probe sets	PPLR values
1.0e−5	957	<7.66e−5	209	<1.17e−4
1.0e−4	1,417	<0.00060	265	<0.000899
1.0e−3	2,196	<0.0059	340	<0.011

### Gene ontology analysis

**Fig. 2:**
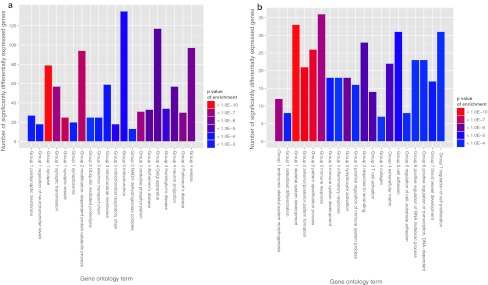
Numbers of significantly differentially expressed genes assigned to enriched gene ontology terms. The 20 most enriched KEGG pathways and GO terms in the categories of biological process and cell component are shown. Redundant terms were removed for clarity. **a** Genes upregulated in oculomotor neurons (*q* value for enrichment <0.05). *Group 1* synaptic function, *Group 2* ubiquitin-mediated proteolysis, *Group 3* oxidative phosphorylation and mitochondrial function, *Group 4* other. **b** Genes upregulated in spinal motor neurons (*q* value for enrichment <0.005). *Group 1* skeletal development, *Group 2* embryonic pattern formation, *Group 3* immune system processes, *Group 4* the extracellular matrix, *Group 5* cell adhesion, *Group 6* regulation of transcription, *Group 7* other

Gene ontology (GO) analysis was performed to determine the functional categories over-represented amongst significantly differentially expressed genes. A significant proportion of genes expressed at higher levels in OM neurons belonged to the functional clusters of synaptic transmission, ubiquitin-mediated protein degradation, and mitochondrial oxidative phosphorylation (Fig. 2a; Table 3). Genes involved in all stages of ubiquitin-mediated proteolysis, and genes with mitochondrial functions, including the respiratory chain complex were upregulated in OM neurons (Supplementary tables 1 and 2). Three KEGG pathways relevant to neurodegenerative diseases (Huntington’s disease, Alzheimer’s disease and Parkinson’s disease) were also enriched amongst genes upregulated in OM neurons. Although ALS was not an enriched functional category, there was upregulation in OM neurons of the expression of a number of genes in which mutations are known to cause motor neuron degeneration, including C9ORF72 (FC 2.56, *q* = 4.0E−6), BSCL2 (FC 3.09, *q* = 0.003), optineurin (FC 2.0, *q* = 0.001), spastin (FC 2.4, *q* = 2.95E−6), and VAPB (FC 1.8, *q* = 1.0E−5).

**Table 3:** Gene ontology enrichment analysis

	Gene ontology term	Count	*P* value	*q* value	Fold enriched
*GO terms enriched in genes upregulated in oculomotor neurons*					
1. Synaptic function	GO:0045202—synapse	79	2.60E−14	1.32E−10	2.50
	GO:0008021—synaptic vesicle	25	1.91E−08	2.42E−05	3.70
	GO:0007268—synaptic transmission	57	1.98E−07	0.00012	2.07
	GO:0045211—postsynaptic membrane	27	0.000125	0.015	2.25
	GO:0019717—synaptosome	20	0.00013	0.015	2.65
	GO:0001505—regulation of neurotransmitter levels	18	9.25E−05	0.012	2.90
2. Ubiquitin-dependent proteolysis	GO:0019941—modification-dependent protein catabolic process	94	4.57E−08	3.86E−05	1.77
	hsa04120: ubiquitin-mediated proteolysis	25	0.00048	0.043	2.13
3. Mitochondrial function	hsa00190: oxidative phosphorylation	31	2.85E−07	0.00014	2.78
	GO:0005746—mitochondrial respiratory chain	18	2.94E−05	0.0055	3.16
	GO:0005739—mitochondrion	135	4.15E−05	0.0075	1.40
	GO:0031966—mitochondrial membrane	59	8.51E−05	0.012	1.68
	GO:0022900—electron transport chain	25	9.91E−05	0.013	2.37
	GO:0030964—NADH dehydrogenase complex	13	0.00020	0.020	3.48
4. Other	hsa05012: Parkinson’s disease	30	6.85E−07	0.00031	2.73
	GO:0031982—vesicle	97	1.39E−06	0.00050	1.63
	GO:0005794—Golgi apparatus	117	4.68E−06	0.0013	1.51
	GO:0043005—neuron projection	57	5.05E−06	0.0013	1.87
	hsa05010: Alzheimer’s disease	33	5.14E−06	0.0013	2.36
	hsa05016: Huntington’s disease	34	1.68E−05	0.0034	2.20
*GO terms enriched in genes upregulated in spinal motor neurons*					
1. Skeletal system	GO:0001501—skeletal system development	33	1.09E−16	3.17E−13	6.30
	GO:0048704—embryonic skeletal system morphogenesis	12	1.65E−09	6.01E−07	12.83
	GO:0001649—osteoblast differentiation	8	4.69E−06	0.00031	11.61
2. Ant/post specification	GO:0009952—anterior/posterior pattern formation	21	1.14E−13	1.66E−10	9.14
	GO:0007389—pattern specification process	26	1.62E−12	1.57E−09	5.93
3. Immune response	GO:0006955—immune response	36	1.97E−09	6.37E−07	3.18
	GO:0046649—lymphocyte activation	18	2.76E−08	7.31E−06	5.51
	GO:0042110—T cell activation	14	1.39E−07	2.18E−05	6.77
	GO:0009611—response to wounding	28	1.52E−07	2.21E−05	3.22
	GO:0002684—positive regulation of immune system process	16	8.57E−06	0.000509	4.10
	GO:0002520—immune system development	18	2.88E−06	0.000215	3.97
	GO:0006954—inflammatory response	18	2.46E−05	0.00116	3.37
4. Extracellular matrix	GO:0031012—extracellular matrix	22	2.42E−07	3.36E−05	3.86
	GO:0005581—collagen	7	2.00E−05	0.000971	12.12
5. Cell adhesion	GO:0007155—cell adhesion	31	1.21E−06	0.000113	2.70
	GO:0010810—regulation of cell-substrate adhesion	8	8.79E−06	0.000512	10.60
6. Transcription	GO:0045893—positive regulation of transcription, DNA-dependent	23	1.15E−05	0.000598	2.94
	GO:0051254—positive regulation of RNA metabolic process	23	1.32E−05	0.000663	2.91
7. Other	GO:0001568—blood vessel development	17	2.68E−06	0.000205	4.23
	GO:0042127—regulation of cell proliferation	31	1.26E−05	0.000644	2.40

The 20 most enriched KEGG pathways and gene ontology terms in the categories of biological process and cell component are shown for each motor neuron subtype. Redundant terms were removed for clarity. *P* value and *q* value apply to the fold enrichment

In LSC motor neurons, upregulated genes were enriched for the functions of skeletal system development, anterior/posterior pattern formation, immune system processes, the extracellular matrix, cell adhesion, and transcription (Fig. 2b; Table 3). Transcriptional regulators expressed at a higher level in LSC motor neurons included 17 of the 39 homeobox (Hox) genes which operate distinct genetic programs along the anterior/posterior axis (Supplementary table 3), and MALAT1, a non-coding RNA recently identified as a principle binding target of TDP43, and thought to play a role in the recruitment of splicing factors [55], which is 8.7-fold upregulated in LSC motor neurons relative to OM neurons (*q* value <1.0E−17). Homeobox genes engrailed 1 and 2, which are known molecular markers of midbrain tissue [5] were upregulated in OM neurons. Extracellular matrix components expressed at higher levels in LSC motor neurons included seven different collagen subunits (Supplementary table 4).

The majority of gene ontology categories enriched in OM and LSC-upregulated genes identify functions which are known to play a role in the pathogenesis of ALS. It was not possible within the scope of this study to explore the potential role that each functional category may play in the relative resistance of OM neurons to the degenerative process. We focussed on the differences observed in the most highly enriched functional category of synaptic transmission.

### Differential expression of genes with a function in synaptic transmission

#### Glutamate-mediated neurotransmission

A number of glutamate receptor subunits were upregulated in OM neurons (Table 4). The AMPA glutamate receptor subtype consists of four subunits, GluR1–GluR4, and the presence of the GluR2 subunit determines the calcium permeability of the receptor. In the absence of GluR2, the AMPA receptor–ion channel complex becomes permeable to calcium. In this study, we showed upregulation of the GluR2 subunit in resistant OM neurons, relative to the vulnerable lumbar motor neuron population. The GluR1 subunit was also expressed at higher levels, as were the synaptic scaffolding proteins, GRIP1 and 2, which interact with GluR2 and GluR3 to stabilize these receptor subunits at the synaptic membrane [7]. There was no differential expression of NMDA receptors.

**Table 4:** Upregulated glutamate receptor subunits and transporters in human oculomotor neurons

Gene name	Symbol	Probe id	Fold change	PPLR value	*q* value
Glutamate receptor, ionotropic, AMPA 1	GLUR1	209793_at	4.82	3.49E−15	0
		211520_at	3.20	5.60E−06	6.59E−07
Glutamate receptor, ionotropic, AMPA 2	GLUR2	205358_at	2.31	1.14E−05	1.36E−06
		236538_at	3.31	0.002008	0.00031
Glutamate receptor, ionotropic, kainate 2	GRIK2	213845_at	5.34	5.09E−07	4.74E−08
Glutamate receptor, metabotropic 3	GRM3	205814_at	2.00	3.58E−08	3.42E−09
Glutamate receptor, metabotropic 7	GRM7	207548_at	2.50	2.30E−06	2.72E−07
Glutamate receptor interacting protein 1	GRIP1	235957_at	2.57	6.37E−06	7.33E−07
Glutamate receptor interacting protein 2	GRIP2	216481_at	3.80	3.19E−09	2.23E−10

#### GABA-mediated neurotransmission

**Fig. 3:**
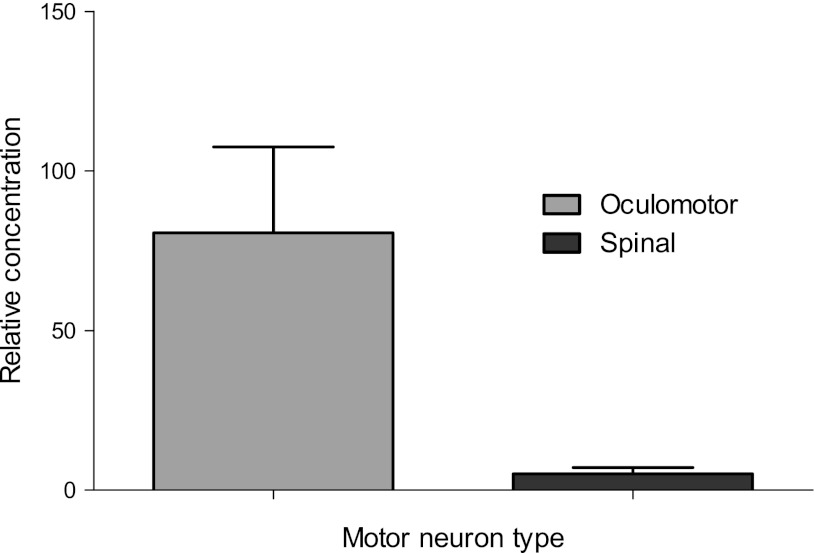
Concentration of GABRA1 relative to β-actin in laser-captured motor oculomotor and spinal motor neurons determined by QPCR (2 cases, *n* = 6 replicates). *Error bars* represent SEM

GABA is the most widely distributed inhibitory neurotransmitter in the mammalian CNS. It signals through GABA_A_ ligand-gated chloride channels, composed of different combinations of 16 subunits, and GABA_B_ metabotropic receptors, which are heterodimers of subunits 1 and 2. In OM neurons, there was upregulation relative to LSC motor neurons, of six GABA_A_ receptor subunits and of GABA_B_ receptor subunit 2 (Table 5). The differential expression of GABRA1 was confirmed using quantitative PCR (Fig. 3). Relative concentration of GABRA1 was 16-fold higher in OM neurons compared to LSC motor neurons, when determined by QPCR.

**Table 5:** Upregulated GABA receptor subunits and genes in human oculomotor neurons

Gene name	Symbol	Probe id	Fold change	PPLR value	*q* value
Gamma-aminobutyric acid (GABA) A receptor, alpha 1	GABRA1	244118_at	7.56	1.16E−07	1.08E−08
Gamma-aminobutyric acid (GABA) A receptor, beta 1	GABRB1	207010_at	5.21	5.35E−08	4.57E−09
Gamma-aminobutyric acid (GABA) A receptor, beta 2	GABRB2	155712 _at	8.76	3.78E−20	0
		242344_at	9.21	2.61E−05	3.21E−06
Gamma-aminobutyric acid (GABA) A receptor, epsilon	GABRE	204537_at	8.18	1.54E−08	1.62E−09
Gamma-aminobutyric acid (GABA) A receptor, gamma 1	GABRG1	241805_at	2.79	7.27E−05	9.25E−06
Gamma-aminobutyric acid (GABA) receptor, theta	GABRQ	238123_at	26.3	1.13E−08	9.84E−10
Gamma-aminobutyric acid (GABA) B receptor, 2	GABBR2	209990_at	3.10	4.93E−09	4.30E−10
		211679_at	2.33	4.83E−06	5.75E−07

#### Verification of microarray findings by QPCR and in other species

Limited motor neuron numbers in the oculomotor nucleus precluded the verification of differential expression by QPCR in a large panel of genes. We therefore aimed to confirm the changes found in human tissue, using microarray datasets from two other species—rat and mouse, which were obtained from a public repository. These studies were performed on whole oculomotor nucleus and whole spinal cord, and the gene expression profiles obtained are therefore from a more heterogeneous cell population than that of our human study, which was highly enriched for motor neurons, due to the laser capture process. Nevertheless, the differential gene expression profiles obtained from mouse and rat oculomotor nucleus versus spinal cord, and those from human OM neurons versus LSC motor neurons were highly correlated. On gene ontology analysis, only 2 of the top 20 GO terms enriched amongst genes upregulated in OM neurons and LSC motor neurons, respectively, were unique to the human study, the others all being significantly enriched in the mouse and/or rat study. In both other species, functional categories relating to synaptic transmission were the most highly enriched in differentially expressed genes (Supplementary table 5). Homologues of 12 of the 16 GABA and glutamate receptor subunits that were significantly differentially expressed between human OM neurons and LSC motor neurons were also significantly differentially expressed between oculomotor nucleus and spinal cord in mouse and/or rat tissue (Table 6).

**Table 6:** Significantly upregulated glutamate and GABA receptor subunits and genes in mouse and rat oculomotor nucleus

Gene name	Symbol	Mouse			Rat		
		Probe id	Fold change	*P* value	Probe id	Fold change	*P* value
Glutamate receptor, ionotropic, AMPA 1	GLUR1	1435239_at	2.49	0.0024	1371013_at	2.05	4.79E−06
		1448972_at	1.83	0.0379			
Glutamate receptor, ionotropic, AMPA 2	GLUR2				1387171_at	2.19	4.86E−09
					1368401_at	2.02	4.23E−08
Glutamate receptor, metabotropic 7	GRM7				1369781_at	1.29	0.0011
Glutamate receptor interacting protein 1	GRIP1				1376988_at	1.48	0.0101
Glutamate receptor interacting protein 2	GRIP2				1387954_at	1.29	0.0011
					1388016_at	4.95	2.38E−06
Gamma-aminobutyric acid (GABA) A receptor, alpha 1	GABRA1	1421281_at	8.24	1.98E−04			
		1436889_at	5.62	1.85E−05			
Gamma-aminobutyric acid (GABA) A receptor, beta 1	GABRB1				1369904_at	2.30	3.82E−04
					1369371_at	1.96	1.42E−08
					1388030_at	1.71	5.67E−04
					1388039_at	1.42	9.54E−07
					1375720_at	1.39	6.49E−07
Gamma-aminobutyric acid (GABA) A receptor, beta 2	GABRB2	1450319_at	2.39	0.0059	1369818_at	2.51	1.93E−06
					1387383_at	1.64	3.11E−05
					1368952_at	1.38	0.00285
Gamma-aminobutyric acid (GABA) A receptor, epsilon	GABRE				1388049_at	1.84	3.19E−05
Gamma-aminobutyric acid (GABA) A receptor, gamma 1	GABRG1				1387706_at	1.25	0.0300
Gamma-aminobutyric acid (GABA) receptor, theta	GABRQ				1387706_at	1.25	0.0300
Gamma-aminobutyric acid (GABA) B receptor, 2	GABBR2				1370701_at	1.63	0.0279

All GABA and glutamate receptor subunits differentially expressed in human oculomotor and spinal motor neurones are included in the table, and data from rat and mouse oculomotor nucleus versus spinal cord shown, where significant differential expression was found

#### Glutamate and GABA receptor-mediated currents in lumbar spinal cord and oculomotor neurons in slice preparations from rat

**Fig. 4:**
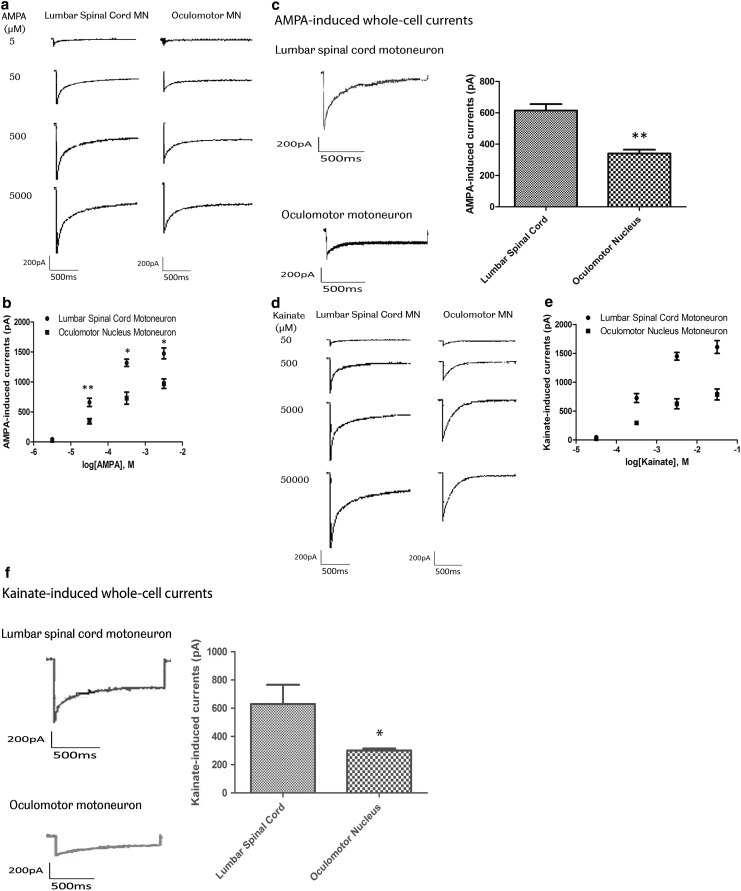
**a** and **b** Concentration–response relation for AMPA-induced whole-cell currents in lumbar spinal cord and oculomotor neurons. Currents were recorded in 20 mM extracellular Na^+^ at −60 mV, in response to AMPA concentrations ranging from 5 μM to 5 mM. **a** Representative current traces elicited by AMPA. **b** Each point represents mean ± SEM from three cells (*P < 0.05, **P < 0.01, Student’s *t* test). EC_50_ values estimated from fits to pooled data were 118.6 μM for lumbar spinal cord motor neurons and 123.2 μM for oculomotor neurons. **c** Whole-cell currents recorded in Na^+^-free extracellular solution containing 50 mM Ca^2+^ at −60 mV in lumbar spinal cord and oculomotor neurons, evoked by AMPA 100 μM (*n* = 6). *Columns* represent the peak amplitudes of agonist-induced whole-cell currents (mean ± SEM, **P < 0.001, *P* < 0.05, Student’s *t* test). **d** and **e** Concentration–response relation for kainate-induced whole-cell currents in lumbar spinal cord and oculomotor neurons. Currents were recorded in 20 mM extracellular Na^+^ at −60 mV, in response to kainate concentrations ranging from 50 μM to 50 mM. **d** Representative current traces elicited by kainate. **e** *Each point* represents mean ± SEM from three cells (*P < 0.05, **P < 0.01, Student’s *t* test). EC_50_ values estimated from fits to pooled data were 1.12 mM for lumbar spinal cord motor neurons and 1.26 mM for oculomotor motor neurons, respectively. **f** Whole-cell currents recorded in Na^+^-free extracellular solution containing 50 mM Ca^2+^ at −60 mV in lumbar spinal cord and oculomotor neurons, evoked by kainate 1 mM (*n* = 6). *Columns* represent the peak amplitudes of agonist-induced whole-cell currents (mean ± SEM, **P < 0.001, *P* < 0.05, Student’s *t* test)

**Fig. 5:**
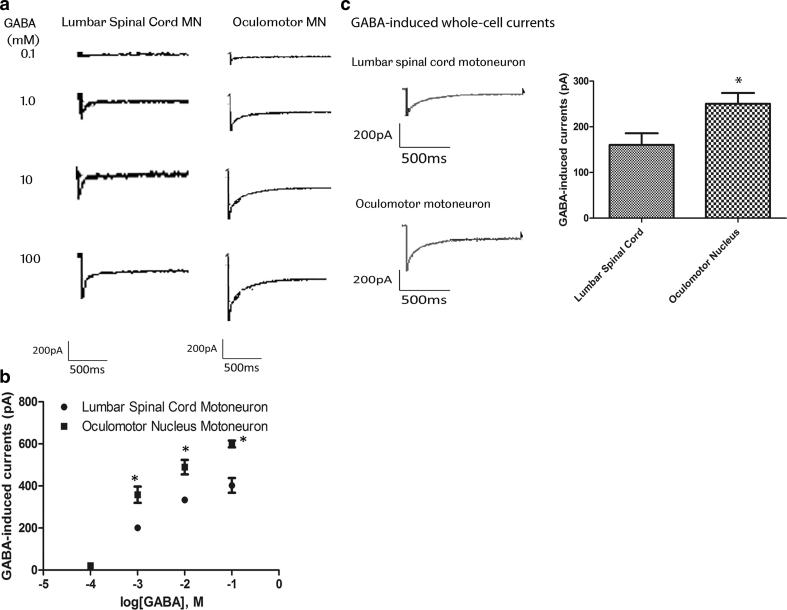
**a** and **b** Concentration–response relation for GABA-induced whole-cell currents in lumbar spinal cord and oculomotor motor neurons. Currents were recorded at −60 mV, in response to GABA concentrations ranging from 0.1 mM to 100 mM. *Each point* represents mean ± SEM from three cells (*P < 0.05, Student’s *t* test). EC_50_ values estimated from fits to pooled data were 6.1 mM for lumbar spinal cord motor neurons and 5.7 mM for oculomotor motor neurons, respectively. **c** GABA-induced whole-cell currents in lumbar spinal cord and oculomotor neurons. Whole-cell currents evoked by 6 mM GABA were recorded at −60 mV. *Columns* represent the peak amplitudes of GABA-induced whole-cell currents (mean ± SEM, *P < 0.05, *n* = 6, Student’s *t* test)

The functional significance of the observed changes in glutamate and GABA receptor subunits was determined using patch clamp recording of whole-cell currents in slice preparations of the rat lumbar spinal cord and midbrain. To assess AMPA receptor-mediated whole-cell currents, three cells were used for dose–response recordings for AMPA- and kainate-induced whole-cell currents in OM and LSC motor neurons. 100 μM AMPA and 1 mM kainate were used to measure Ca^2+^ permeability of AMPA receptors in six cells per agonist. Both AMPA- and kainate-induced currents were significantly larger in LSC motor neurons than in OM neurons. AMPA-induced currents were 614.00 ± 102.21 pA in LSC motor neurons and 340.67 ± 58.80 pA in OM neurons. Kainate-induced currents were 629.50 ± 334.57 in LSC motor neurons and 300.50 ± 34.22 in OM neurons (Fig. 4). To assess GABA receptor-mediated whole-cell currents, three cells were used for dose–response recording for GABA. 10 mM GABA was used for repeat recording in six cells. Amplitudes of whole-cell currents evoked by 10 mM GABA were significantly smaller in LSC motor neurons than in OM neurons. GABA-induced currents were 160.33 ± 62.43 pA in LSC motor neurons and 250.33 ± 58.03 pA in OM neurons (Fig. 5).

## Discussion

We have identified marked differences in the transcriptional profiles of human spinal motor neurons, which are susceptible, and oculomotor neurons, which are resistant to the degenerative process in ALS. Nearly 2,000 genes are significantly differentially expressed by the two motor neuron subtypes, with a conservative false discovery rate of 0.001. This is the first study of differences in the gene expression characteristics between ALS-resistant and ALS-vulnerable motor neuron subtypes in human tissue. A recent paper by Hedlund et al. [14] used microarray analysis of motor neurons of normal mice, to compare the transcriptional profiles of OM, hypoglossal and cervical spinal motor neurons. They also found differential expression between genes in the functional categories of mitochondrial function, ubiquitination, cell adhesion and RNA metabolism. Upregulation of Hox genes was seen in cervical spinal motor neurons and hypoglossal neurons, compared to oculomotor neurons. However, three genes highlighted in the study, IGF-1 and guanine deaminase (upregulated in oculomotor neurons), and peripherin (upregulated in spinal motor neurons), showed no differential expression in our study of human motor neurons, which may reflect species-specific differences in expression between the motor neuron subtypes.

### Functional categories of genes differentially expressed between oculomotor and spinal motor neurons

Using Gene Ontology enrichment analysis, we have shown that motor neuron subtypes with different susceptibility to degeneration in ALS show differential expression of genes with a function in synaptic transmission, ubiquitin-dependent proteolysis, mitochondrial function, transcriptional regulation, immune system functions and the extracellular matrix. These differences may reflect their diverse embryological origin, the different milieu in the brainstem, or differences in the structure and function of oculomotor units compared to motor units of other skeletal muscles. For example, upregulation of genes with mitochondrial functions, including the respiratory chain complex, is likely to reflect the high metabolic demand placed on oculomotor neurons, due to their high firing rate. Hox genes are key controllers of rostro-caudal patterning, and of motor neuron pools within each level of the hindbrain and spinal cord [3, 4, 11]. The patterns of differential expression of Hox genes and of engrailed 1 and 2 therefore reflect the positions of these neurons on the rostro-caudal axis. The functional implications of differential expression of collagen subunits between OM and LSC motor neurons are unknown, but are intriguing in the light of findings of altered composition of the extracellular matrix in patients with ALS, and the clinical observation that they are resistant to bedsores [24, 42, 43].

Amongst the differences in gene expression observed, are transcriptional characteristics that render oculomotor neurons resistant to the degenerative process in ALS. For example, there is evidence that the capacity of the proteasome system to degrade proteins may be a limiting factor in the vulnerability of neurons to the degenerative process [19]. Our observation of an increase in the expression of genes encoding components of the ubiquitin–proteasome system in OM neurons compared to LSC motor neurons is a novel finding, which may contribute to their relative resistance to degeneration. We have made this array data freely available to the scientific community (http://www.ncbi.nlm.nih.gov/geo/; GSE 40438) to explore other gene expression changes which may lead to a functional difference between OM and LSC motor neurons.

We used datasets from two different species, obtained from the GEO Omnibus repository, to confirm the differential gene expression seen on microarray analysis in human OM and LSC motor neurons. There was marked concordance in the functional categories of genes differentially expressed between the three species analysed, with the most significant enrichment occurring in all three species, in genes with a function in synaptic transmission. GABA and glutamate receptor subunits differentially expressed between the motor neuron subtypes in human tissue showed concordant changes in mouse and/or rat tissue in the majority of cases.

### Relative resistance of oculomotor neurons to excitotoxicity

There is substantial evidence that excitotoxicity plays a role in the motor neuronal death of ALS, and that motor neurons are selectively vulnerable to excitotoxic cell injury. Motor neurons possess atypical AMPA receptors that express proportionately fewer GluR2 receptor subunits, relative to other GluR subunits [22, 53]. This low expression of GluR2 would render them more permeable to calcium, and thus more vulnerable to excitotoxic injury. Spinal motor neurons show a higher AMPA receptor current density than ALS-resistant dorsal horn neurons [57]. SOD1^G93A^ transgenic mice crossed with ChAT-GluR2 mice that overexpress the GluR2 subunit in cholinergic neurons have reduced calcium permeability of the motor neuronal AMPA receptors, and show prolonged survival compared to the single heterozygote SOD1^G93A^ mice [54]. GluR2 pre-mRNA is edited by the enzyme ADAR2 (adenosine deaminase acting on RNA2), to convert adenosine to inosine at the Q/R site. Failure of this editing function has been described in motor neurons of patients with sporadic ALS [21]. Furthermore, conditional knockdown of ADAR2 in motor neurons of mice [15], or expression of a functionally modified GluR2 subunit with increased calcium permeability [25] causes a slow decline in motor neurons of the spinal cord and brainstem. In ADAR2 knockout mice, this motor neuron degeneration spares the oculomotor nucleus, suggesting that excitotoxicity is an important determinant of the selective vulnerability of spinal motor neurons, a finding that is further supported by the present study.

We observed upregulation in OM neurons relative to LSC motor neurons of several GABA and glutamate receptor subunits. A previous study showed no difference between ALS-resistant and -vulnerable brainstem motor nuclei in the expression of mRNA or protein of GluR2 and GluR4 [26]. However, we found that OM neurons express higher levels of GluR2, which may render them less susceptible to excitotoxic injury. Although the proportional expression of GluR2 relative to other GluR receptor subunits was not determined in this study, our functional data confirm that the expression changes observed lead to a higher level of calcium influx in response to AMPA/kainate in spinal motor neurons than in oculomotor neurons. The higher level of expression of GABA receptor subunits in OM neurons may reflect increased expression of GABA receptors, which may be neuroprotective through increasing GABA transmission to protect against excessive neuronal excitability. Alternatively, the upregulation of some GABA receptor subunits in OM neurons may reflect alteration in subunit composition, which is thought to determine functional diversity of the receptor [28]. This finding is supported by an earlier study showing lower expression of GABAR subunits in vulnerable than resistant motor neurons in brainstem motor nuclei of normal rats [31].

We carried out patch clamp recording of AMPA and GABA-induced whole-cell currents in OM and LSC motor neurons from rat, in order to determine the functional effect of the observed differences in expression of AMPA and GABA receptor subunits. Amplitudes of whole-cell currents evoked by AMPA receptor agonists were dramatically larger in LSC motor neurons than OM neurons. This difference in AMPA receptor current between the two types of motor neuron, observed with both AMPA and kainate as agonists, was detected over the full range of the agonist concentration–response curves. These whole-cell currents were generated by inward influx of calcium ions, through AMPA receptor activation, as they were observed in extracellular solutions free of Na^+^, and in the presence of MK-801 to block NMDA receptors, tetrodotoxin to block voltage-gated Na^+^ channels and Cd^2+^ to block voltage-gated Ca^2+^ channels. The higher amplitude of the receptor-mediated current in LSC motor neurons could be explained by a higher density of AMPA receptors, but this is not supported by the microarray data, which showed reduced expression of AMPA receptor subunits in LSC motor neurons. Alternatively, the differential expression of GluR1 and GluR2 subunits may reflect an alteration in subunit composition, which could result in an increase in single-channel conductance or in open probability of calcium channels. A relatively higher somatic size in spinal motor neurons could also result in higher amplitude of the receptor-mediated current, but this is unlikely to explain the differences observed, as the GABA_A_-induced current was decreased in LSC motor neurons relative to OM neurons. Amplitudes of GABA_A_ receptor-mediated inward chloride currents were significantly higher in OM neurons than in LSC motor neurons. This higher amplitude could be explained by a higher expression of GABA receptors in OM neurons, reflected by the observed increase in GABRA1, B1 and B2 receptor subunit expression, or by altered subunit composition of the receptors, resulting in a change in the physiological channel properties.

The difference in AMPA and GABA-mediated inward currents, with an increase in AMPA-mediated inward calcium current, and decrease in GABA-mediated inward current in LSC motor neurons, relative to OM neurons, would be predicted to increase the susceptibility of spinal motor neurons to excitotoxicity. Interest in excitotoxicity in ALS has largely focussed on the role of excess glutamate activity; however, insufficient synaptic inhibition may also play an important role. Spinal motor neurons and oculomotor neurons are innervated by GABAergic inhibitory neurons [35, 51], which generate fast inhibitory potentials to regulate the amplitude and duration of excitatory postsynaptic potentials. GABAergic transmission has been shown to be neuroprotective in motor neuron cultures exposed to glutamate [58], and in other experimental paradigms where excitotoxicity contributes to neuronal death [34]. Loss of cortical inhibitory signals [8], and depletion of cortical GABAergic interneurons [37] have been demonstrated in patients with ALS. PET studies have also shown reduction in binding of the GABA_A_ receptor ligand, flumazenil, in the extramotor cortex of ALS patients, correlating with the extent of cognitive dysfunction [30]. Furthermore, patients with rapidly progressive disease were shown to have loss of cortical inhibitory GABAergic mechanisms, compared to those with a more slowly progressive ALS variant [56]. There has been increasing interest recently in the potential role of degeneration of inhibitory interneurons in the pathogenesis of ALS. Reduced expression of GABAergic and glycinergic markers has been demonstrated in the ventral horns, and reduced glycine-mediated currents in cultured motor neurons from mutant SOD1 mice [2, 16]. The present study contributes to a growing body of evidence that a reduction in GABA-mediated synaptic transmission plays a role in the pathogenesis of selective degeneration of spinal motor neurons in ALS.

## Conclusion

We have used genome-wide transcriptome analysis to determine systematically the differences between two populations of motor neurons that show differential susceptibility to degeneration in ALS. The transcriptional profiles of oculomotor and spinal motor neurons are distinct, with differences in nearly 2,000 genes, at a conservative false discovery rate. Significant differences might be expected due to the different embryological origin, location, and structure and function of oculomotor units. However, amongst the changes observed are those that determine the disease-resistant properties of oculomotor neurons. Analysis of the transcriptional profiles of oculomotor nucleus and spinal cord tissue of two further species showed that differential expression of genes with a function in synaptic transmission, including GABA and glutamate receptor subunits, is conserved across the three species. We carried out functional studies, using patch clamp recording to measure whole-cell currents induced by AMPA and GABA receptor agonists, and found significant upregulation of AMPA-induced calcium currents, and downregulation of GABA-induced chloride currents in the disease-susceptible spinal motor neurons. These microarray and electrophysiology data provide strong evidence that reduced susceptibility to excitotoxicity, mediated in part through increased GABAergic transmission, is an important determinant of the relative resistance of oculomotor neurons to degeneration in ALS.

## Electronic supplementary material

Below is the link to the electronic supplementary material.

Supplementary Figures (DOC 787 kb)

Supplementary experimental procedures (DOC 25 kb)

Supplementary Table 1 (XLS 125 kb)

Supplementary Table 2 (XLS 211 kb)

Supplementary Table 3 (XLS 100 kb)

Supplementary Table 4 (XLS 68 kb)

Supplementary Table 5 (DOCX 24 kb)

## Footnotes

1 Bioconductor is an open-source computational framework for the analysis of high-throughput genomic data implemented in the R programming language.
