# Supplementary material for: Unravelling the enigma of selective vulnerability in neurodegeneration: motor neurons resistant to degeneration in ALS show distinct gene expression characteristics and decreased susceptibility to excitotoxicity
Source: Acta Neuropathol. 2012 Nov 13;125(1):95–109. doi: 10.1007/s00401-012-1058-5 (PMC3535376; doi:10.1007/s00401-012-1058-5)
Supplement: Supplementary file 1 — Supplementary Figures (DOC 787 kb) [file 401_2012_1058_MOESM1_ESM.doc]

# Supplementary figures

# Supplementary figure 1


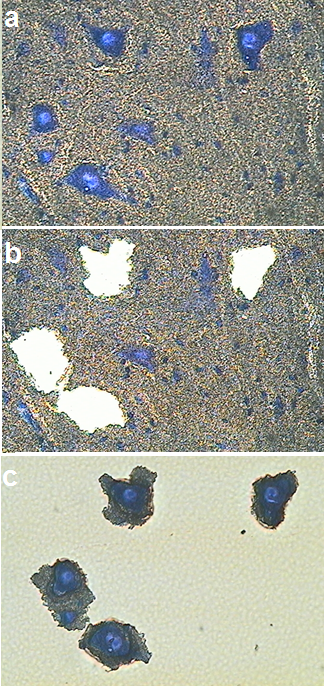


Laser capture microdissection: a) Tissue section through anterior horn of spinal cord with toluidine-blue stained motor neurons b) Post laser capture c) Motor neurons isolated on laser capture cap.

# Supplementary figure 2


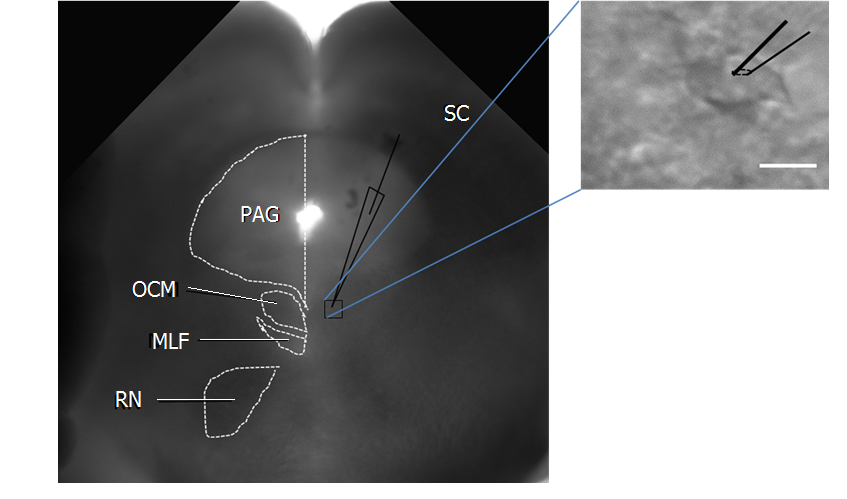


Photomicrograph of a transverse section showing the location of oculomotor nucleus: SC, superior colliculus; PAG, periaqueductal grey; OCM, oculomotor nucleus; MLF, medial longitudinal fasciculus; RN, red nucleus. Scale bar=20µM.
