# Supplementary material for: Unravelling the enigma of selective vulnerability in neurodegeneration: motor neurons resistant to degeneration in ALS show distinct gene expression characteristics and decreased susceptibility to excitotoxicity
Source: Acta Neuropathol. 2012 Nov 13;125(1):95–109. doi: 10.1007/s00401-012-1058-5 (PMC3535376; doi:10.1007/s00401-012-1058-5)
Supplement: Supplementary file 2 — Supplementary experimental procedures (DOC 25 kb) [file 401_2012_1058_MOESM2_ESM.doc]

# Supplementary experimental procedures

# Buffers for patch clamp recording:

1) Bicarbonate buffered saline: 118mM NaCl, 3mM KCl, 1mM MgCl_2_, 25mM NaHCO_3_, 1mM NaH_2_PO_4_, 1.5mM CaCl_2_, 20mM glucose at pH 7.4

## 2) Intracellular solution for AMPA / Kainate induced currents: 120mM CsF, 3mM MgCl2, 5mM EGTA, and 10mM HEPES (pH adjusted to 7.25 with 12 mM CsOH)

## 3) Intracellular solution for GABA induced currents: 140mM CsCl, 10mM HEPES, 10mM 1,2-*bis*(2-aminophenoxy)ethane-N,N,N',N'-tetraacetic acid (BAPTA); 2mM Na2-adenosine 5'-triphosphate (ATP) _4_, MgCl_2_, pH 7.25.

## 4) Extracellular perfusion buffer for AMPA / Kainate dose response: 15.3mM NaCl, 4.7mM NaOH, 2mM CaCl_2_, 10mM HEPES, 10mM, and 228mM sucrose, pH 7.40

## 5) Sodium-free extracellular perfusion buffer for measurement of calcium permeability of AMPA receptors: 47.8 mM CaCl_2_, 2.2 mM Ca(OH)_2_, 10 mM glucose, 10 mM HEPES, and 147 mM sucrose, pH 7.4.

## 6) Extracellular perfusion buffer for GABA dose response: 125mM NaCl, 3.5mM KCl, 1.3mM MgSO4, 1.2mM KH2PO4, 26mM NaHCO3, 10mM glucose and 2.4mM CaCl2, pH 7.3.
