# Supplementary material for: Unravelling the enigma of selective vulnerability in neurodegeneration: motor neurons resistant to degeneration in ALS show distinct gene expression characteristics and decreased susceptibility to excitotoxicity
Source: Acta Neuropathol. 2012 Nov 13;125(1):95–109. doi: 10.1007/s00401-012-1058-5 (PMC3535376; doi:10.1007/s00401-012-1058-5)
Supplement: Supplementary file 7 — Supplementary Table 5 (DOCX 24 kb) [file 401_2012_1058_MOESM7_ESM.docx]

**Supplementary table 5: Gene ontology terms enriched in genes significantly differentially expressed, in human, mouse and rat.**

The 20 most enriched KEGG pathways and gene ontology terms in the categories of biological process and cell component are shown, with q value <0.05. Redundant terms were removed for clarity. FE = Fold Enrichment; p-value and q-value apply to the fold enrichment. KEGG pathways are *homo sapiens* specific, and not applicable to mouse and rat datasets.

**Oculomotor neurons**

| **GROUP** |  | **Human** | | | | |  | **Mouse** | |  | **Rat** | |
| --- | --- | --- | --- | --- | --- | --- | --- | --- | --- | --- | --- | --- |
| **1. Synaptic function** |  | **Term** | **Count** | **p-value** | **q-value** | **FE** |  | **p value** | **FE** |  | **p value** | **FE** |
|  |  | GO:0045202~synapse | 79 | 2.60E-14 | 1.32E-10 | 2.50 |  | 5.76E-10 | 3.96 |  | 2.66E-32 | 2.74 |
|  |  | GO:0008021~synaptic vesicle | 25 | 1.91E-08 | 2.42E-05 | 3.70 |  | 5.68E-08 | 8.06 |  | 1.43E-12 | 3.47 |
|  |  | GO:0007268~synaptic transmission | 57 | 1.98E-07 | 0.00012 | 2.07 |  | 0.003809 | 2.80 |  | 2.24E-21 | 2.94 |
|  |  | GO:0045211~postsynaptic membrane | 27 | 0.000125 | 0.015 | 2.25 |  | 0.0002 | 4.01 |  | 8.53E-14 | 2.90 |
|  |  | GO:0019717~synaptosome | 20 | 0.00013 | 0.015 | 2.65 |  |  |  |  | 1.12E-07 | 2.52 |
|  |  | GO:0001505~regulation of neurotransmitter levels | 18 | 9.25E-05 | 0.012 | 2.90 |  | 0.016 | 4.02 |  | 1.62E-11 | 3.34 |
| **2. Ubiquitin dependent proteolysis** |  | GO:0019941~modification-dependent protein catabolic process | 94 | 4.57E-08 | 3.86E-05 | 1.77 |  |  |  |  | 4.30E-07 | 1.84 |
|  |  | hsa04120:Ubiquitin mediated proteolysis | 25 | 0.00048 | 0.043 | 2.13 |  | n/a | n/a |  | n/a | n/a |
| **3. Mitochondrial function** |  | hsa00190:Oxidative phosphorylation | 31 | 2.85E-07 | 0.00014 | 2.78 |  | n/a | n/a |  | n/a | n/a |
|  |  | GO:0005746~mitochondrial respiratory chain | 18 | 2.94E-05 | 0.0055 | 3.16 |  |  |  |  |  |  |
|  |  | GO:0005739~mitochondrion | 135 | 4.15E-05 | 0.0075 | 1.40 |  |  |  |  | 2.08E-12 | 1.49 |
|  |  | GO:0031966~mitochondrial membrane | 59 | 8.51E-05 | 0.012 | 1.68 |  |  |  |  | 2.81E-06 | 1.65 |
|  |  | GO:0022900~electron transport chain | 25 | 9.91E-05 | 0.013 | 2.37 |  |  |  |  | 7.45E-04 | 2.21 |
|  |  | GO:0030964~NADH dehydrogenase complex | 13 | 0.00020 | 0.020 | 3.48 |  |  |  |  |  |  |
| **4. Other** |  | hsa05012:Parkinson's disease | 30 | 6.85E-07 | 0.00031 | 2.73 |  | n/a | n/a |  | n/a | n/a |
|  |  | GO:0031982~vesicle | 97 | 1.39E-06 | 0.00050 | 1.63 |  | 0.000113 | 2.27 |  | 9.82E-13 | 1.75 |
|  |  | GO:0005794~Golgi apparatus | 117 | 4.68E-06 | 0.0013 | 1.51 |  | 0.020484 | 1.60 |  | 2.18E-09 | 1.60 |
|  |  | GO:0043005~neuron projection | 57 | 5.05E-06 | 0.0013 | 1.87 |  | 3.89E-07 | 3.78 |  | 1.11E-27 | 2.46 |
|  |  | hsa05010:Alzheimer's disease | 33 | 5.14E-06 | 0.0013 | 2.36 |  | n/a | n/a |  | n/a | n/a |
|  |  | hsa05016:Huntington's disease | 34 | 1.68E-05 | 0.0034 | 2.20 |  | n/a | n/a |  | n/a | n/a |

**Spinal motor neurons**

| **GROUP** |  | **Human** | | | | |  | **Mouse** | |  | **Rat** | |
| --- | --- | --- | --- | --- | --- | --- | --- | --- | --- | --- | --- | --- |
| **1. Skeletal system** |  | **Term** | **Count** | **p-value** | **q-value** | **FE** |  | **p value** | **FE** |  | **p value** | **FE** |
|  |  | GO:0001501~skeletal system development | 33 | 1.09E-16 | 3.17E-13 | 6.30 |  | 5.76E-10 | 3.965601 |  | 6.31E-10 | 2.05 |
|  |  | GO:0048704~embryonic skeletal system morphogenesis | 12 | 1.65E-09 | 6.01E-07 | 12.83 |  | 3.78E-11 | 5.171492 |  | 1.76E-04 | 2.50 |
|  |  | GO:0001649~osteoblast differentiation | 8 | 4.69E-06 | 0.00031 | 11.61 |  | 0.003809 | 2.802878 |  | 4.78E-04 | 2.65 |
| **2. Ant / post specification** |  | GO:0009952~anterior/posterior pattern formation | 21 | 1.14E-13 | 1.66E-10 | 9.14 |  |  |  |  |  |  |
|  |  | GO:0007389~pattern specification process | 26 | 1.62E-12 | 1.57E-09 | 5.93 |  |  |  |  |  |  |
| **3.**  **Immune response** |  | GO:0006955~immune response | 36 | 1.97E-09 | 6.37E-07 | 3.18 |  |  |  |  | 3.22E-08 | 1.72 |
|  |  | GO:0046649~lymphocyte activation | 18 | 2.76E-08 | 7.31E-06 | 5.51 |  |  |  |  | 8.73E-04 | 1.70 |
|  |  | GO:0042110~T cell activation | 14 | 1.39E-07 | 2.18E-05 | 6.77 |  | 0.000113 | 2.274935 |  | 6.25E-04 | 1.91 |
|  |  | GO:0009611~response to wounding | 28 | 1.52E-07 | 2.21E-05 | 3.22 |  | 1.56E-07 | 6.081859 |  | 1.00E-17 | 2.13 |
|  |  | GO:0002520~immune system development | 18 | 2.88E-06 | 0.000215 | 3.97 |  | 0.020484 | 1.607561 |  | 6.40E-05 | 1.66 |
|  |  | GO:0002684~positive regulation of immune system process | 16 | 8.57E-06 | 0.000509 | 4.10 |  | 3.89E-07 | 3.786474 |  | 2.21E-07 | 1.93 |
|  |  | GO:0006954~inflammatory response | 18 | 2.46E-05 | 0.00116 | 3.37 |  |  |  |  | 1.30E-05 | 1.80 |
| **4. Extracellular matrix** |  | GO:0031012~extracellular matrix | 22 | 2.42E-07 | 3.36E-05 | 3.86 |  |  |  |  | 1.21E-19 | 2.59 |
|  |  | GO:0005581~collagen | 7 | 2.00E-05 | 0.000971 | 12.12 |  |  |  |  | 1.60E-03 | 3.27 |
| **5. Cell adhesion** |  | GO:0007155~cell adhesion | 31 | 1.21E-06 | 0.000113 | 2.70 |  |  |  |  | 4.56E-10 | 1.77 |
|  |  | GO:0010810~regulation of cell-substrate adhesion | 8 | 8.79E-06 | 0.000512 | 10.60 |  |  |  |  | 3.79E-03 | 2.37 |
| **6. Transcription** |  | GO:0045893~positive regulation of transcription, DNA-dependent | 23 | 1.15E-05 | 0.000598 | 2.94 |  |  |  |  | 1.35E-02 | 1.28 |
|  |  | GO:0051254~positive regulation of RNA metabolic process | 23 | 1.32E-05 | 0.000663 | 2.91 |  |  |  |  | 1.65E-02 | 1.27 |
| **7. Other** |  | GO:0001568~blood vessel development | 17 | 2.68E-06 | 0.000205 | 4.23 |  | 2.35E-05 | 2.610372 |  | 1.24E-13 | 2.38 |
|  |  | GO:0042127~regulation of cell proliferation | 31 | 1.26E-05 | 0.000644 | 2.40 |  |  |  |  | 2.10E-20 | 1.95 |
